# Serious Financial Problem and Suggested Remedy

**Published:** 1920-03-06

**Authors:** 


### THE LONDON HOSPITAL.

### Serious Financial Problem and Suggested Remedy.

AT a meeting of the Quarterly Court, held on Wednesday, the financial outlook of the London Hospital was laid before the Governors. Particulars were given of the work of the past year. In-patients reached the enormous figure of 19,699, and out-patients 118,685. These out-patients made no less than 450,000 attendances. The work has become unmanageably heavy, and a Committee is to sit, composed of members of the Committee and members of the staff, to decide what is to be done to reduce or to meet this overwhelming pressure. There are 837 patients waiting for admission.

But this increasing pressure is not the only difficulty. The serious and heartbreaking difficulty is to find the money to meet this mass of work. So far the subscriptions have not been reduced. But letters from subscribers are received which say that this is the last time they can subscribe. This is partly due to the uncertainty as to what the Ministry of Health proposes to do with the voluntary hospitals. Partly, also, of course, to the growing difficulties of the middle class to make both ends meet.

### GROWING COST OF TREATMENT.

But with this threatened reduction of income there is another problem to be faced. This is that the cost of treatment is growing week by week. Most difficult and costly examinations have to be made to get at accurate diagnosis. Rule-of-thumb work has gone. Half a dozen departments focus their attention on a patient—*x*-rays, bacteriological, chemical, sterilising, and so on. No patient is treated by one man, but by a team of experts. If disease has to be prevented as well as cured, that must be so. So that at a time when increased income is needed the hospital is met with the danger of a fall in income and an ever-growing expenditure.

There is a debt to the bank of £52,000. There are securities to sell to meet that debt, but no hospital can go on for long selling its securities. There are several methods of action :

(1) To make a big appeal to the generosity of the public. But too many sister hospitals are doing that already.

(2) To close half the hospital, and go on closing, bed by bed, as income falls. But what, then, is to become of the sick poor of East London and the waiting-list at 837?

(3) To appeal to the Government for help. Everybody is doing that. Possibly the amusement tax might be allocated to hospitals.

### SUGGESTED SCHEME OF PATIENTS' PAYMENTS.

The only suggestion that the Committee can make is that every patient admitted shall pay something towards his keep. The cost of every patient is £3 17s. per week.

It is suggested that every patient be asked to contribute at least 10s. a week towards the cost—10s. out of £3 17s. is not much. It does not even pay for the food a patient takes. The medical, surgical, and nursing service will still be given. One thing, however, is certain : the hospital cannot go on longer on the present lines.

Within the last few weeks the Committee has had to refuse several schemes for the improvement of certain departments which would make the work done in them all the more efficient, and all for the reason of lack of funds. It has been admitted in each case that the improvement was necessary.

### STATISTICS OF FIFTY YEARS.

In fifty years the expenditure has increased from £29,000 to £200,000, the beds in use from 431 to 805, and the in-patients treated from 4,800 to 19,700.

The death rate has fallen from 11 per cent. in 1868 to 7 per cent. in 1918. But this is no sort of measure of increasing efficiency, because nowadays the hospital admits and cures patients it would not have dreamed of admitting at all in 1868. They were at that time incurable. Now they are not. There are incurable diseases now which also will be curable. It is heart-breaking that progress is barred at this time. Science is on the point of making most important life-saving discoveries. The best brains in the world, here and elsewhere, are at work at cancer, for instance, and at a time when there is opportunity of fighting disease instead of fighting our fellow-men all is held up for lack of money.

### MEDICAL AND SURGICAL UNITS.

A scheme whereby a physician and a surgeon shall be appointed, not in an honorary capacity, but to a paid post and giving the whole of their time to the care of patients and to the study of disease with a view to its prevention, has been approved by the Governors. The Committee advertised the appointments, and applications were received not only from all parts of the country, but from America and Canada.

They recommend that Dr. Charles Miller be appointed head of the medical unit, and Mr. H. S. Souttar head of the surgical unit. It is hoped that great advances will be made in the knowledge and treatment of disease and improving the education of the doctor of the future.

